# A Misdiagnosed Complication of a Metal-on-Metal Total Hip Arthroplasty

**DOI:** 10.1155/2018/6413814

**Published:** 2018-03-15

**Authors:** Az-Eddine Djebara, Cédric Joseph, Florence Rousseau, Benoit Brunschweiler, Patrice Mertl

**Affiliations:** ^1^Department of Orthopaedics and Traumatology, CHU Amiens Picardie, 80480 Amiens, France; ^2^Department of Infectious Diseases, CHU Amiens Picardie, 80480 Amiens, France; ^3^Department of Bacteriology, CHU Amiens Picardie, 80480 Amiens, France

## Abstract

**Introduction:**

The enterobacterial genus *Yersinia* includes a number of human pathogens. Large-diameter, metal-on-metal prostheses are no longer used because of their high failure rate. Here, we describe the first case of *Yersinia enterocolitica* infection of a metal-on-metal total hip arthroplasty.

**Clinical Examination:**

A metal-on-metal prosthesis failed ten years after implantation. After surgical revision, bacteriological testing revealed the presence of a pathogenic strain of *Yersinia enterocolitica*. Combination antibiotic therapy resulted in a favorable clinical outcome.

**Discussion:**

Three cases of hip arthroplasty infected with *Yersinia enterocolitica* have been described in the literature. The present case is the first infection of a metal-on-metal total hip arthroplasty. We suggest that the risk of infection is increased by the release of metal wear particles and their influence on the surrounding tissue.

**Conclusion:**

When a large-diameter, metal-on-metal total hip arthroplasty fails, the known complications associated with this type of prosthesis should not deter the physician from screening for an infectious process that requires specific treatment.

## 1. Introduction

Three members of the enterobacterial genus *Yersinia* (*Yersinia enterocolitica*, *Yersinia pestis*, and *Yersinia pseudotuberculosis*) are human pathogens. *Yersinia enterocolitica* (*YE*) is occasionally responsible for acute intestinal infections (especially in young patients) with secondary joint and dermatological symptoms. The digestive symptoms usually disappear after 1 to 3 weeks. In immunosuppressed patients, however, the infection can persist and cause bacteraemia. The joint symptoms usually appear two weeks after the digestive signs and are usually due to aseptic, reactive arthritis. A few cases of septic arthritis with *Yersinia* in native joints and on prostheses have been described in the literature [1–3]. Here, we report on the first case of *YE* infection of a large-diameter, metal-on-metal total hip arthroplasty (THA).

## 2. Clinical Findings

**Figure 1:**
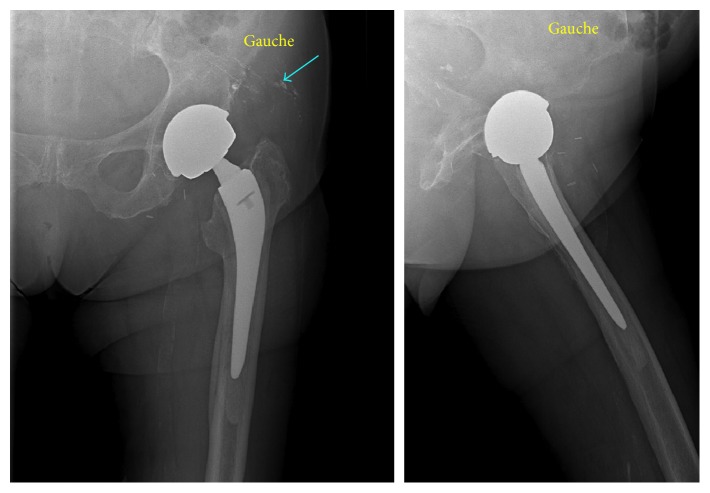
Left metal-on-metal THA with a pseudocyst.

**Figure 2:**
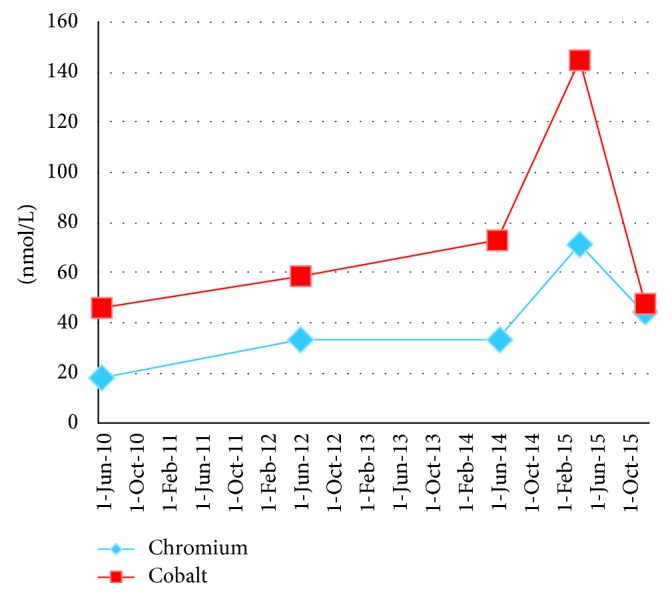
Change in serum chromium and cobalt levels over time.

**Figure 3:**
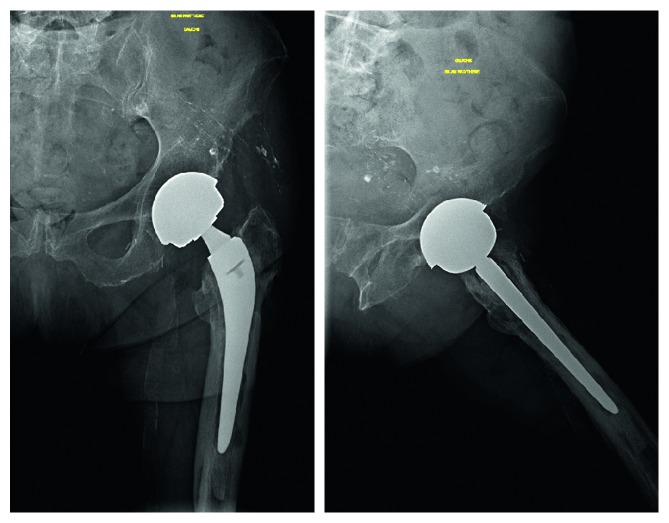
Endosteal osteolysis around the stem.

**Figure 4:**
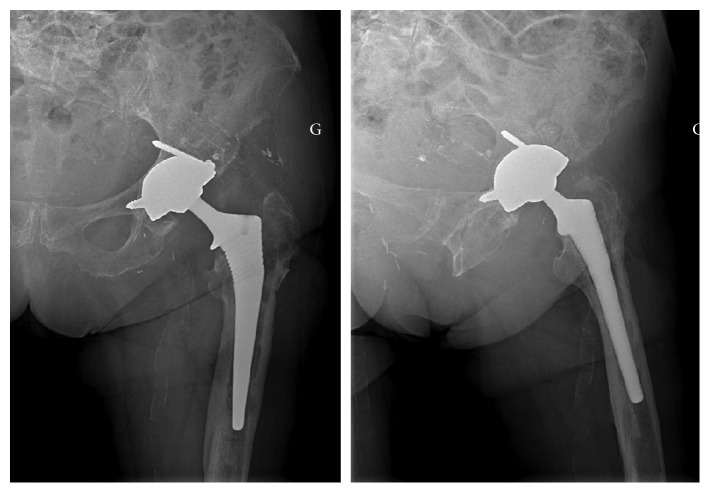
Postoperative X-ray.

An 85-year-old patient received regular checkups for a left, large-diameter, metal-on-metal THA (a Contact® stem and a Durom® cup) implanted 11 years previously. His medical history included right THA in 1989 (revised in 1995) and mucosa-associated lymphoid tissue lymphoma of the left eye (treated with surgery and chemotherapy). Laboratory assays showed abnormally high but stable serum levels of chromium (33 nmol/L) and cobalt (73 nmol/L) during follow-up (normative values: <10 nmol/L for both metals). Following the onset of severe pain in the left hip, imaging (X-rays, ultrasound, and CT) revealed a calcified pseudocyst (Figure 1), a frequent complication of metal-on-metal prostheses [4]. The serum chromium and cobalt levels had risen to 71 nmol/L and 145 nmol/L, respectively (Figure 2), and appeared to be correlated with the patient's pain levels, although the precise cause of the pain remained unclear [5]. The pain intensity continued to increase, whereas the serum C-reactive protein level was stable (110 mg/l). Three months later, endoscopy exploration of the digestive tract revealed a hiatal hernia. Biopsies of the fundus and duodenum were negative. Colonoscopy revealed sigmoid diverticulitis, and two polyps were removed. The patient was reexamined 6 months later. A hip X-ray showed marked endosteal osteolysis around the stem (Figure 3). The serum chromium and cobalt levels had fallen to 43 nmol/L and 48 nmol/L, respectively. We decided to replace the prosthesis and proceeded to implant a cementless stem and a dual mobility cup (Figure 4). During surgery, we discovered a voluminous pseudocyst containing milky fluid and encapsulated by a pseudomembrane. Eight deep-tissue samples were sent for histopathological assessment and bacteriological testing. The results confirmed the presence of inflammation around the pseudosynovial membrane, with a macrophage reaction around metal wear particles.

## 3. Bacteriology

Direct examination of Gram-stained samples was negative. After 48 hours of culture on selective growth media and colony picking, mass spectrometry confirmed the presence of *YE*. In parallel, Gram staining revealed small, Gram-negative, urease-positive bacilli. Antibiotic susceptibility testing showed that the bacterium was sensitive to third-generation cephalosporins, aminoglycosides, trimethoprim + sulfamethoxazole, and tetracycline, intermediate for quinolones, and resistant to ampicillin, amoxicillin + clavulanic acid, ureidopenicillins, and carboxypenicillins. The bacterium was later sent to the French National *Yersinia* Reference Centre (Pasteur Institute, Paris) for pathogenicity screening; biochemical tests and serotyping confirmed that the *YE* strain was potentially pathogenic in humans. Following a multidisciplinary team meeting (with the surgeon, an infectious disease specialist, and a medical biologist), probabilistic, broad-spectrum, dual-antibiotic therapy was initiated immediately after replacement of the implant (cefepime and vancomycin for 2 days, followed by trimethoprim + sulfamethoxazole 800 mg three times a day and ceftriaxone 1.5 g twice a day for 6 weeks).

## 4. Clinical Outcome

The postoperative course was uneventful, and the patient was able to walk immediately. He was discharged from hospital nine days after surgery. At the time of writing (six months later), he is able to walk unaided at home, the scar is clean, and there are no signs of osteolysis or displacement of the implant on X-rays. One month after the discontinuation of antibiotic therapy, the patient was free of pain, and his serum CRP level had normalized.

## 5. Discussion

*Yersinia* infections outside the digestive tract are rare but usually result from blood dissemination from the gut in immunocompromised subjects [6]. There are very few studies of septic complications of metal-on-metal THA [7, 8]. Three cases of THA infection with *YE* have been described in the literature. Hansen et al. reported the first case of septic loosening of an Austin Moore prosthesis implanted with an indication of osteonecrosis [1]. Hougaard and Søgaard described a chronic THA infection ten years after surgery [2]. Jean-Pierre et al. reported on a second case of septic loosening [3]. Although friction torque appears to be associated with an abnormally high infectious complication rate, it is difficult to establish a clear correlation. It has been hypothesized that high concentrations of metal wear particles, metal ions, and corrosion products have molecular effects on the surrounding soft tissues and thus change the local environment [9, 10] Paradoxically, it seems that the Co-Cr particles released into dynamic growth media by metal-on-metal THAs inhibit biofilm formation in some germs—making them more vulnerable to clearance by the immune system or antibiotics [11]. In the present case, we suspect that endoscopic investigation of the digestive tract during the patient's course of chemotherapy caused blood dissemination of the bacteria. However, in an interview, the patient did not recall experiencing any digestive problems in the weeks preceding surgery. The onset of this infection (discovered fortuitously during surgery) cannot therefore be determined with accuracy.

## 6. Conclusion

When a large-diameter, metal-on-metal THA fails, the known complications associated with this type of prosthesis should not deter the physician from screening for an infectious process. A *YE* infection may be present and will require specific antibiotic treatment, along with surgical revision.
